# Classification of cancer cells at the sub-cellular level by phonon microscopy using deep learning

**DOI:** 10.1038/s41598-023-42793-9

**Published:** 2023-09-27

**Authors:** Fernando Pérez-Cota, Giovanna Martínez-Arellano, Salvatore La Cavera, William Hardiman, Luke Thornton, Rafael Fuentes-Domínguez, Richard J. Smith, Alan McIntyre, Matt Clark

**Affiliations:** 1 Optics and Photonics Group, Faculty of Engineering, University of Nottingham, Nottingham, UK; 2 Institute for Advanced Manufacturing, Faculty of Engineering, University of Nottingham, Nottingham, UK; 3 Biodiscovery Institute, Centre for Cancer Sciences, School of Medicine, University of Nottingham, Nottingham, UK

**Keywords:** Biomedical engineering, Biophysics, Cancer, Applied optics

## Abstract

There is a consensus about the strong correlation between the elasticity of cells and tissue and their normal, dysplastic, and cancerous states. However, developments in cell mechanics have not seen significant progress in clinical applications. In this work, we explore the possibility of using phonon acoustics for this purpose. We used phonon microscopy to obtain a measure of the elastic properties between cancerous and normal breast cells. Utilising the raw time-resolved phonon-derived data (300 k individual inputs), we employed a deep learning technique to differentiate between MDA-MB-231 and MCF10a cell lines. We achieved a 93% accuracy using a single phonon measurement in a volume of approximately 2.5 μm^3^. We also investigated means for classification based on a physical model that suggest the presence of unidentified mechanical markers. We have successfully created a compact sensor design as a proof of principle, demonstrating its compatibility for use with needles and endoscopes, opening up exciting possibilities for future applications.

## Introduction

Cancer is one of the biggest causes of death in the developed world. Globally, cancer was the cause of death for nearly 10 million people in 2020^1^. The predicted 10-year survival rate after detection is typically only 50%^2^, so there is a significant clinical need to gain a better understanding of cancer and improve diagnostics and therapy. One aspect of cancer discovery that has gained significant interest lately, is the role that stiffness plays in the process of tumorigenesis^3–5^. Palpation is one historical and simple diagnostic technique that makes use of the mechanobiology of some types of cancer. Distinct differences in mechanobiology also have been detected both at the single cell and macroscopic tumour scales, among normal, primary, and metastatic conditions^3,5,6^. Therefore, the contrast obtained through the characterisation of elasticity could contribute to addressing some of the challenges of cancer: identifying cancerous tissue and quantifying the malignant potential of individual cancer cells in-vivo is key to early diagnosis. This is where optics alone often struggle to differentiate normal cells/tissue from their cancerous counterpart and, more importantly, correlate the observed differences with mechanisms on cancer-associated phenotypes.

Considerable advancements have been made in the assessment and comprehension of cancer's elastic properties at the single-cell level^7,8^. Although atomic force microscopy has been predominantly utilised in most studies^9–11^, alternative techniques, including microfluidics^12^, micropipette aspiration^13^, optical tweezers^14^ and ultrasound^15,16^ have also been employed. It has been widely agreed upon that cancer cells exhibit lower elasticity compared to normal cells^17^ enabling their discrimination^18–20^. However, challenges persist in fully understanding these disparities and effectively applying them for cancer diagnosis in a clinical setting. These obstacles primarily stem from limitations in throughput, with most studies ignoring sub-cellular detail, as well as the inherent difficulties associated with conducting in-vivo measurements.

Phonon microscopy^21–23^ is a highly promising technique for the examination of elastic properties at the single-cell level. Its contrast is given by the sound velocity, its lateral resolution is given by optics and exhibits super-optical axial resolution. All these features arise due to the ability of phonon microscopy to generate and detect the time of flight of coherent phonons via Brillouin scattering^22^. The collection of Brillouin scattering techniques has also enabled significant growth in several techniques used for the high-resolution characterisation of elasticity^21,24,25^. Different modalities of spontaneous^24,26^, stimulated^27^ and time-resolved^28–30^ Brillouin scattering have demonstrated great potential for three-dimensional elasticity mapping at optical^31^ and super-optical resolutions^23^. For instance, recent reports have highlighted applications in cancer^32,33^, parasitology^34^ and plant biology^31^.

Along with new contrast modalities, artificial intelligence is one new tool that has been proposed for overcoming challenges with the optical classification of cancer. It is expected that artificial intelligence will accelerate discovery by assisting, enhancing, and streamlining the use of the available data. Diagnosis^35,36^, drug discovery^37^, and genetics^38^ have all benefited from the use of artificial intelligence. For example, deep learning models have been trained on optical images of different types of cancer and normal cells in a flow system with accuracies ranging from approximately 90–99%^39^. Another study used artificial intelligence to classify dead and living breast cancer cells with and without treatment to accuracies of over 94%^40^.

This article demonstrates the use of phonon microscopy^21,22,41^ (a time-resolved Brillouin scattering technique), and artificial intelligence to distinguish between normal and cancerous breast cells based on their elasticity properties. An unprecedentedly large phonon-based dataset (with 300 k individual time-resolved phonon measurements) was acquired where the estimated sound velocity and attenuation from the data reveal a trend for softer cancer cells. Using the data to train a convolutional neural network (CNN) model, we achieved the classification with information gathered in a 2.5 μm^3^ volume. The model was further tested using simulated data which suggests the presence of common biomarkers across cell lines. Finally, we present preliminary needle-based measurements with the footprint required for future translation to clinical applications.

## Results

**Figure 1:**
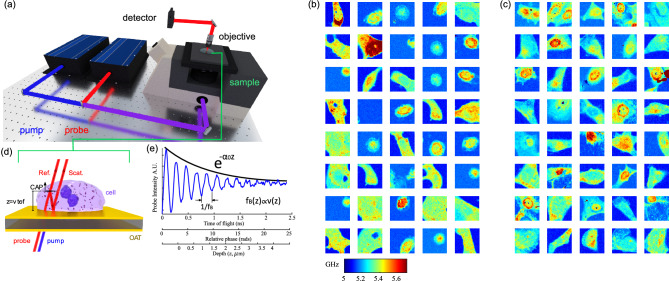
Phonon microscopy measurements and the time-resolved signal. (**a**) Simplified schematic of the experimental setup for phonon microscopy: pump and probe light are delivered through an inverted microscope to an optoacoustic transducer (OAT), while the transmitted probe light is collected by a secondary objective lens. (**b**) Brillouin frequency maps of normal breast cells (MCF10a, normal control). (**c**) Brillouin frequency maps of cancer breast cells (MDA-MB-231, cancer). These maps represent a simplified version of the main dataset utilized in this study. The fundamental frequency was extracted from each time-resolved signal (one per pixel) to generate the Brillouin frequency maps. (**d**) Light-sound interactions within the cell give rise to time-resolved Brillouin scattering. (**e**) Representative time-resolved signal where the time of flight reveals the axial position and speed of the phonon wavefront through changes in the relative phase between the reference and scattered beams.

Phonon microscopy^21–23,41^ (see Fig. 1a) uses Brillouin scattering as means of producing images with contrast based on elasticity (refer to Fig. 1d). Unlike Brillouin microscopy which employs incoherent phonons, phonon microscopy generates and detects coherent phonons to obtain a time-resolved signal (see Fig. 1e). The frequency of the signal is the Brillouin frequency shift which is a function of the sound velocity within the volume being imaged and is used to create the qualitative elasticity maps shown in Fig. 1b,c. The signal also exhibits an exponential decay which lends to a measurement of the sound attenuation coefficient α_0_ (see Fig. 1e). The sound velocity and attenuation coefficient relate to the stiffness and viscosity of the material under examination. The time of flight of the signal produced by phonon microscopy (refer to Fig. 1e) enables sectioning of the volume in the axial dimension with resolution determined by the phonon wavelength, (approximately 300 nm)^23^. Thereby the time-resolved signal enables 3D imaging without requiring further scanning. This capability, to the best of our knowledge, has not been used for cancer cell classification.

We captured images of both normal control (MCF10a) and cancerous (MDA-MB-231) breast cells using phonon microscopy. In total, we measured 80 cells (40 of each type), as depicted in Fig. 1b,c. These maps were created primarily for visualization purposes, by determining the fundamental frequency of each signal (giving the colour to each pixel) across time, thus providing an average measurement across the volume. Each cell was imaged using 61 × 61 measurements summing up to nearly 300,000 individual time-resolved signals and 16 times as many independent voxels (of ~ 0.15 μm^3^, see “Materials and methods”).

**Figure 2:**
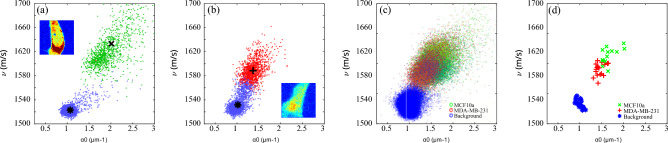
Clustering of the signal parameters extracted from the experimental data. (**a**,**b**) Examples of clustering from the data set. There is a good distinction with the background obtained by frequency thresholding. (**c**) Scatter plot for all the individual measurements of 24 cells from the data set. The individual measurements in (**a**–**c**) are presented with a transparency. (**d**) Centroids of each class for the same cells as (**c**). Accurate discrimination from this analysis would be challenging in an in-vivo scenario.

We extracted the sound velocity (ν) and attenuation coefficient (α_0_) by performing a fit (see “Materials and methods”) to assess our ability to discriminate the signals using their measurable physical parameters. This allowed us to generate a scatter plot where each occurrence in the plot is coloured according to the type of cell: normal in green (MCF10a), cancerous in red (MDA-MB-231) and background in blue (saline buffer). The cells were segmented by frequency thresholding to separate them from their backgrounds (see section “Discriminating cancer and normal cells using deep learning” below). Figure 2a,b shows the scatter plots from two cells of the dataset with their centroids indicated. The cell and background clusters are well separated according to their sound velocity and attenuation coefficient. However, the scatter plots from 12 normal and 12 cancer cells shown in Fig. 2c overlap significantly. This overlap can arise from a number of reasons including variations in the cell cycle, the close similarity in the cell lines (benign and malignant tumour) as well as the fixation process.

By determining the centroids for each cell individually (see Fig. 2d), a trend becomes apparent and shows that, on average, cancer cells exhibit lower sound velocity and sound attenuation compared to normal cells. Lower sound velocity and attenuation generally indicate lower elastic modulus (softer material) which has been previously observed in several works^17^.

### Discriminating cancer and normal cells using deep learning

Discrimination between cancerous and normal cells through cluster analysis in an in-vivo or in-vitro scenario would be a difficult task due to the prolonged time it would take to obtain the necessary measurements. In this approach, fitting the time-resolved signal to a sinusoidal function causes critical information, contained within the instantaneous phase of the signal, to be disregarded. This includes all the phase changes given by the variation of the sound velocity in depth as well as sharp phase transitions caused by structural features. These features are challenging to identify and extract using conventional signal processing as the signal is usually a complicated mix of components generated by sound waves travelling in different directions from different interfaces. As it remains unclear which specific elastic features may contribute to cancer/normal discrimination, it is important not to make any assumptions about what these features may be. For this reason, rather than handcrafting features, as is done in classical machine learning, a deep learning approach was taken to exploit automatic feature extraction capabilities working directly with the raw data.

**Figure 3:**
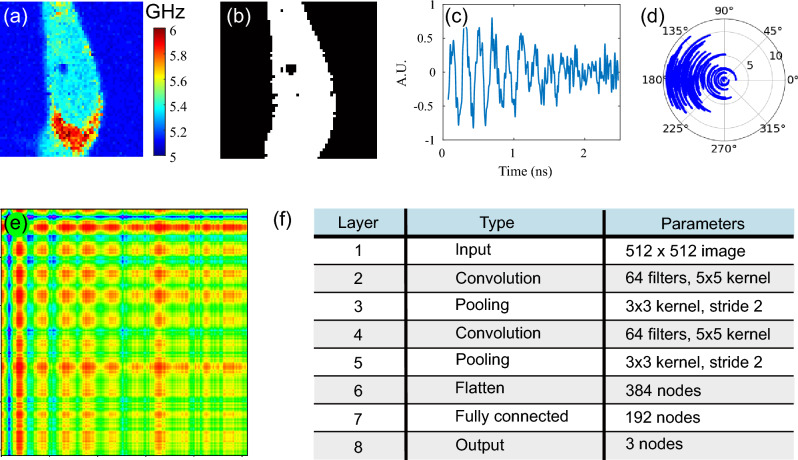
Data preparation. (**a**) Example of a frequency map of a cell which is used to generate a mask (**b**) to separate the surrounding medium from the cell which provides means for labelling. (**c**) Example of time trace corresponding to a single pixel of the image in (**a**). (**d**) Conversion of (**c**) into polar coordinates. (**e**) Input image for the CNN generated from (**c**) using Gramian angular summation field (**f**) The shallow CNN architecture consists of two convolutional layers and three classes. The label of the image can be *normal*, *abnormal*, or *background* depending on its position in the image and the type of cell being imaged.

The phonon measurements from each scan were separated based on whether these represented cell culture medium or cells depending on their Brillouin frequency (see Fig. 3a,b). This resulted in three classes: *normal* (MCF10a breast cell), *abnormal* (MDA-MB-231, cancer breast cell) and *background* (no cell). The 80 cells, split among four batches (from four experimental sessions), were randomised with an equal number of observations taken from each batch to remove bias from non-relevant contributions to the signal through the common *background* class. For instance, environment-driven fluctuations in laser power, temperature, and transducer characteristics can generate bias.

All the time signals (see Fig. 3c) have the same acquisition characteristics and were converted to an image using the Gramian angular summation fields method^42^ as shown in Fig. 3c–e (see “Materials and methods”). This generated nearly 300,000 images, one corresponding to each time-resolved signal and which were labelled according to the signal location and cell type as explained above. We used a convolutional neural network (CNN, see Fig. 3f) with a softmax activation function and Adam gradient descent optimiser. This is a broadly used and well-established approach for the classification of image data sets^43^. We have chosen a LeNet-based^44^ architecture due to its shallow characteristics allowing us to start understanding if there are changes between normal and cancer cells in high-level features of the signal that cannot be extracted through conventional signal processing.

The CNN was trained using 70% of the dataset, making sure there was an equal number of inputs from the two cell types across all classes. The resultant model was tested with the remaining 30% of cells. By using a signal-by-signal methodology, we were able to eliminate the influence of morphology and focus on elasticity-related information. This involves classifying each measurement individually, and subsequently, the discrimination criteria are based on the measurements performed on a small sub-volume of the cell.

**Figure 4:**
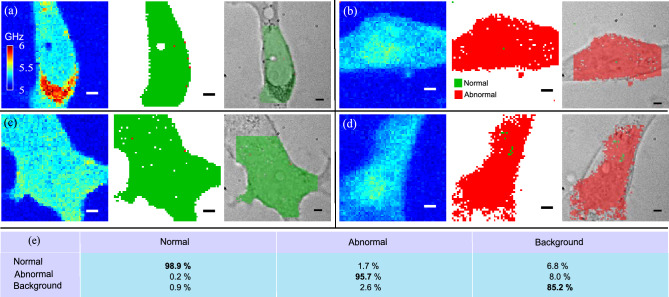
Examples of CNN predictions across complete cells. The class output of the CNN for all the measurements of four cells was put together using the original spatial information. (**a**–**d**) Complete cells from the test dataset where the three subfigures represent left to right: Brillouin frequency maps, CNN-predicted class output and overlay of the optical image and the predicted classification. Control cells (MCF10a) are presented in (**a**,**c**) and cancer cells (MDA-MB-231) in (**b**,**d**). (**e**) A confusion table from 1000 random samples from the test set. All scale bars are 5 μm.

The model classified the Gramian images from the test set with good accuracy (at single measurement/voxel level) with a total 93% accuracy (24 test cells) despite having no laterally resolvable information. Examples of the evaluation of complete cells from the test data are shown in Fig. 4. The classification of the measurements performed on these cells was reconstructed using the spatial coordinates of the original scans. This indicates that the model demonstrates the capability not only to accurately classify cells within a very small volume (as obtained from a single signal, as described in the “Materials and methods”) but also to achieve classification using a considerably lower number of measurements compared to the discrimination presented in Fig. 2.

The confusion table in Fig. 4e, obtained from the test dataset, shows that the medium class has relatively low accuracy at 85%, which could be due to inaccuracies of the masking between cell and background (see Fig. 2b). However, there was low confusion (~ 2%) between the *normal* and *abnormal* classes. By considering these results in terms of sensitivity and specificity for potential diagnosis^45^, we calculated sensitivity and specificity of 0.94 and 0.92 respectively. False *background* were only considered in the calculation of false positives and false negatives since true *background* classifications are inconclusive and neither positive nor negative.

### Probing the means of classification of the CNN model

**Figure 5:**
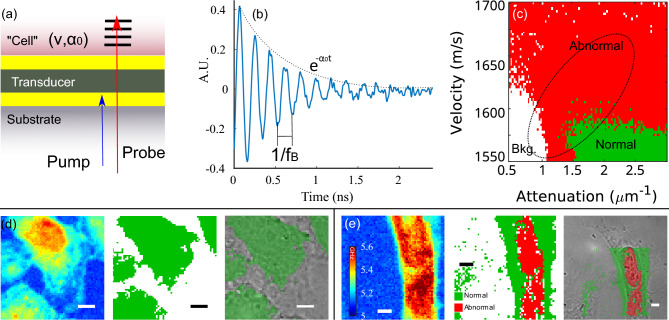
Testing of the AI model using simulation and additional cell lines. (**a**) The geometry of the simulation with an infinite substrate and single “cell” layer. The characteristics of the signal change according to the sound velocity (ν) in the material and the attenuation of rate (α_0_). (**b**) Example of a time trace produced by the model where the sound velocity modifies the signal’s frequency while the attenuation coefficient modifies its decay rate. (**c**) CNN-based classification of the simulated signals for a range of sound velocity and attenuation values. The ellipse represents the data range of the training data shown in Fig. 2 Example 3T3 Fibroblast (**d**) and HeLa (**e**) cells showing (left to right) the Brillouin frequency map, CNN classification and optical picture with CNN classification overlaid. All scale bars are 5 μm.

The application of artificial intelligence raises a question about the specific features that convolutional neural networks have identified and used for data classification. To briefly investigate this, a synthetic dataset was generated using a physical model based on the work of Matsuda et al^46^. This simulation of optomechanical processes allowed us to create a simple homogeneous model of a “cell” (see Fig. 5a). The model geometry consists of a single layer with sound velocity (ν) and sound attenuation coefficient (α_0_). The parameters of this layer were then iterated from 1550 to 1850 m/s for ν and 0.25–3 µm^−1^ for α_0_ to match experimental values previously measured on several cell types^21^. The model used a density of 1050 kg/m^3^^47^, a refractive index of 1.37^48^ and a longitudinal modulus that corresponds to the range of sound velocities. Noise was added afterwards to match the experimental results (see Fig. 5b).

The output of the model (see Fig. 5c) shows clearly defined regions for the classes: low attenuation and low velocity are attributed to the *background* class and high attenuation and low velocity to the *normal* class. The rest of the simulated attenuation-velocity space was attributed to the *abnormal* class (cancer). This simulation explores a range of parameters similar to the experimental observations (see dotted ellipse). The output of the simulation provides an incomplete description of the relationship between cancer, sound velocity and attenuation seen in Fig. 2, yet the model is more effective in achieving the expected classification (93% single-shot accuracy). This suggests that the CNN model is finding unidentified biomarkers that are prevalent in the experimental dataset but are underrepresented in the current simple simulation. Adding further complexity to the physical model in the future can serve as means to identify the nature of such features.

We also conducted classification experiments on two additional cell types that were not included in the original training set: 3T3 mouse fibroblasts and HeLa cells (cervical cancer). These cell types are from different mammalian species or tissues and allow us to assess the level of generalisation of our CNN model. Figure 5d illustrates that the 3T3 fibroblast cells are classified as expected (*normal*) in both the nucleus and cytoplasm regions. For the case of a HeLa cell (see Fig. 5e), the CNN appears to have identified features found in both the *normal* and *abnormal* classes. However, the measurements classified as *abnormal* are spatially correlated with the nucleus of the cell. This strengthens the robustness of the model as it is capable to find features that correlate with the morphology of the cell to which the CNN was not exposed. The result in Fig. 5e also suggests that some of the features identified in the nucleus of MDA-MB-231 may also be present in other types of human cells. This appears plausible, as cancer cells exhibit similar behaviours regardless of the tissue type. This is relevant because indicates the possibility of elasticity as a diagnostics marker that might be valid across multiple cancer tissues. Although this is an exciting proposition, more investigation is required within and across cancer types, subtypes, and normal tissues.

### Phonon diagnostics

So far, we have demonstrated the feasibility of distinguishing between normal and cancerous cell lines derived from the same tissue type. As a result, we suggest that, in the future, this approach could serve as a diagnostic tool in two different ways: first, by conducting phononic-based histopathology analysis on biopsy samples, and second, by utilising optical fibres in an endoscopic setting. Nonetheless, the most significant benefit is likely to arise from in-vivo measurements, which would simplify the biopsy workflow by eliminating the need for tissue extraction and staining.

**Figure 6:**
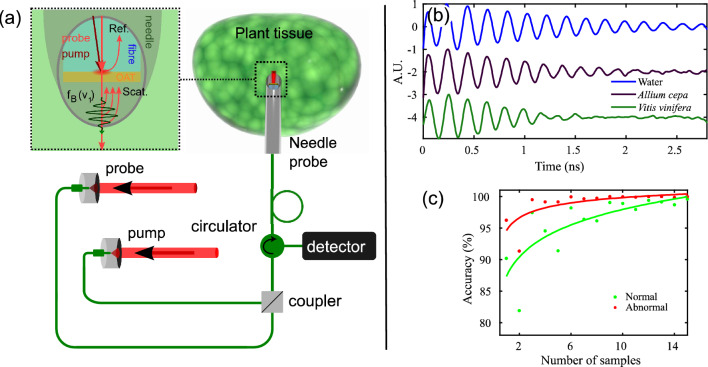
Fibre-probe demonstration and accuracy of multiple measurements. (**a**) Experimental setup of the fibre-based system. The two fibre-coupled laser beams are combined using a coupler and the reference (ref) and scattered (scat) beams are directed to the photodiode using a circulator. (**b**) Exemplar traces from plant specimens (Allium cepa and Vitis vinifera tissue). These measurements show the proof of principle of the application to tissue. (**c**) Increase in classification accuracy of the test set (from Figs. 1, 4) against a number of measurements (for a population of 250). In a fibre-based measuring system, accuracy would increase rapidly by taking multiple spatially resolved measurements by implementing a fibre bundle.

We have recently demonstrated the capability to perform phonon measurements through a single-mode fibre^49^. These fibres are only 125 μm in diameter and compatible with most standard biopsy needle gauges. To explore this further, we have obtained proof of principle measurements using a needle. In this case the samples we used are of plant origin and serve exclusively as an object for demonstration of principle (see Fig. 6). A single-mode fibre was inserted inside a needle with a transducer at its tip (see Fig. 6a). The needle was then approached and inserted into the sample objects to take phonon measurements (see Fig. 6b). The resulting phonon signals show a difference between the tissues (in terms of sound velocity and attenuation) with respect to water and could be processed for data classification as a basis for a diagnosis. However, the fibre probe is currently unable to image in the same way presented in Figs. 1, 4, 5 while inside a needle. Instead, only a limited number of measurements are possible while the needle is inserted into the tissue. However, the accuracy on which cells can be differentiated would still be relatively high.

To improve accuracy further, the single-mode fibre could be replaced with a fibre bundle to perform multiple measurements in parallel as we have demonstrated previously^49^. The number of measurements taken will be increased depending on the number of cores and acquisition speed. Additional measurements will improve accuracy significantly as shown in Fig. 6c: the probability of correct classification increases with the number of samples (from the data and model presented in Figs. 1, 4). The probability was calculated by randomly taking a set number of samples (representing the available number of measurements) 250 times from the test cells to obtain statistics. We can see that at 15 samples (or measurements) an accurate classification is obtained in ~ 98% of the attempts. Therefore, a fibre bundle with 15 cores or more could achieve an accurate classification within approximately 5 s.

Although this seems promising in a clinical setting, there are still many challenges: large amounts of data will be required, and it might be also difficult for the phonon field, at these high frequencies, to reach cells if there are relatively fluid layers around the tissue under examination. Signal generation and retrieval will also be affected when transitioning from single core to bundle as there will be a compromise between coupling tolerances, dispersion and attenuation. We expect the performance and cost of phonon microscopes and probes to improve in the coming years as well as the option to increase the acoustic wavelength to gain imaging depth.

## Discussion

We used phonon microscopy, a technique based on Brillouin scattering, to perform imaging of breast cells, specifically cancerous MDA-MB-231 cells and normal MCF10a cells. The data confirmed previous observations in differences in cancer and normal cells but this time in terms of sound velocity and attenuation. The raw phonon data was employed as inputs for a shallow convolutional neural network, which was trained to distinguish between signals originating from normal cells, cancer cells, and background. To convert the signals into a suitable format for the network, we applied the Gramian angular summation fields method, resulting in image representations of the time-resolved signals. The resulting model achieved an accuracy of 93%. To evaluate its performance, we conducted additional testing through simulations and historical data. Interestingly, we observed a discrepancy between the predictions of the simulated data, which relied on measurable signal features such as frequency and sound attenuation. This suggests that the model may be leveraging unidentified signal features that can potentially serve as biomarkers. Furthermore, we noticed an unexpected consistency in elasticity features across different cell types, confirming the previously observed presence of common characteristics among cancer cells. Lastly, we introduced a crude prototype needle-based sensor capable of capturing the same type of measurement as the phonon microscope, providing a promising future avenue for further research and application.

While the findings of this study hold promising implications for future medical applications, it is crucial to acknowledge the limitations inherent in the current work. Firstly, it is important to note that the dataset used in this study is derived from a relatively small population of cells, comprising a total of 80 samples. To validate and substantiate these observations, a significantly larger and more diverse dataset, encompassing multiple cell lines and types, would be necessary. Furthermore, the cell conditions employed in this study involved growth in a rigid surface, with cell culture media and subsequent fixation in formalin. These conditions differ significantly from the in-vivo environment of cells examined for diagnosis. Crucially, some of the elastic contrast is modified due to crosslinking between formalin and uncharged reactive amino acids. Nevertheless, we provide strong evidence that classification remains feasible following fixation. This might be achievable because of the high frequency (GHz) and resolution (300 nm axially) of our method. Under these conditions, it is likely that sensitivity to localised stiffness and density characteristics of the fixed cells is adequate for discrimination. However, this topic requires further investigation. Moving towards live specimens is expected to provide us with improved elasticity-related contrast (leading to enhanced diagnostic capability). Nonetheless, this transition also presents us with additional challenges. For instance, limited acquisition speed might cause movement artefacts and the act of probing itself could stimulate the cells and affect the measurements. For these reasons, it is necessary to validate these findings using living specimens in future studies.

Nevertheless, there is a need for improvement in conventional biopsy approaches used for the clinical identification of diseases. These approaches involve the extraction and processing of tissue samples, which not only adds cost and time to the diagnostic process but also carries several additional drawbacks. Extracted tissue samples are perishable and prone to contamination, and the extraction process itself can cause wounds to the patient that requires time to heal before repeat procedures can be performed. Furthermore, there is often a need for retesting due to the potential extraction of the wrong region of interest. The overall process is expensive, and the shortage of histopathologists^50^ adds to the challenges faced in this area. It is with this motivation in mind that we aim to leverage the combination of phonon-based contrast, artificial intelligence, and fibre probes to develop a potential endoscopic/needle-based in-vivo cancer diagnostic method.

## Materials and methods

### Phonon microscopy

Phonon microscopy^21^ is a technique that uses laser-generated phonons and Brillouin scattering for imaging and characterisation. The phonons, unlike in spontaneous Brillouin scattering, are coherent and therefore they are generated at the same time travelling in the same direction. This gives rise to the time-resolved signal by interfering the scattered with the non-scattered beam. The phonon microscope uses the pump-probe method around an asynchronous optical sampling (ASOPS) configuration. In this configuration, two pulsed lasers (∼150 fs, 100 MHz repetition rate) are synchronised with a fixed difference in repetition rate. The difference in rate provides means to reconstruct the GHz signal due to the sweeping delay between pulses. The pump pulse is frequency doubled (390 nm, 1 mW) to improve absorption by an optoacoustic transducer (OAT) and facilitate separation. The absorbed light causes rapid heating via thermal expansion and launches coherent phonons into the sample. The probe beam (780 nm, 2 mW) is detected in transmission collected by a secondary objective.

Figure 1a shows a typical experimental configuration of the phonon microscope. We used Menlo systems C-fibre 780 femtosecond erbium pulsed lasers for both probe and pump, objective lenses with NA of 0.6 for light delivery (and brightfield imaging) and 0.42 for light collection. The signal arises due to the interference of the reflected or transmitted probe laser beam with a portion scattered from the acoustic wave packet propagating in the sample (see Fig. 1d). As the wave propagates, the phase of the scattered component changes relative to the non-scattered light producing an oscillating signal commonly referred to as time-resolved Brillouin scattering (TRBS). The detected signal reveals the position (time of flight), relative amplitude and speed (Brillouin frequency) of the phonon field (see Fig. 1e). In this work, we have acquired signals with 5 k averages taking approximately 3 points/s.

Axial resolution for the estimation of the Brillouin frequency is given by^22,23^: z_min_ = λ_probe_/2n, where n is the refractive index of the volume under examination. Axial resolution is set by the phonon wavelength which is independent of the numerical aperture of the system. Considering the 780 nm probe wavelength and using water refractive index we obtain an axial resolution of ~ 300 nm. The lateral resolution is given in this case by the Rayleigh criterion of the phonon generation spot given by the pump laser (x_min_ ~ 400 nm). For a typical propagation of the phonon wavefront of ~ 4 μm (2.5 ns time of flight at the sound velocity of water), we probe a volume of ~ 2.5 μm^3^ and individual voxels of 0.15 μm^3^.

### Needle-based phonon measurements

To demonstrate the proof of principle for phononic measurements of tissue through a biopsy needle, a custom patch cable (780 HP fc/pc connector for proximal termination and bare fibre for distal termination) was fed through a 25G hypodermic needle. With the fibre fed through the needle, the fibre transducer at the bare end was coated with 15 nm of gold using a sputter coater. The light was delivered and collected (using fibre elements, see Fig. 6a) according to the protocols presented in previous works^49^. Using a stereo microscope (10 ×), a small droplet of fast-curing epoxy was placed onto the tip of the needle and retracted the fibre transducer backwards until it was within the bore of the needle making sure its face was not covered with epoxy. The effect of the position of the fibre tip with respect to the needle bore, remains a topic for investigation. The needle was then inserted into the Allium cepa and Vitis vinifera tissue to obtain the measurements.

### Deep learning

Deep learning techniques are data-driven methods that have made major advances in fields such as image recognition^51^, speech recognition^52^ and natural language processing^53^, to name a few. Compared to traditional machine learning techniques, deep learning methods can automatically learn representations (i.e. features) from high-dimensional raw data, without the need for domain expertise or manual feature engineering to perform classification or regression tasks. This is particularly important when the relevant features are unknown and when no assumptions about the data can be made. Deep learning models learn these representations of data with multiple levels of abstraction by composing simple but non-linear modules that each transform the representation at one level, the raw input, into a representation at a higher more abstract level^54^. Through the composition of enough such transformations, a deep learning model can approximate very complex functions. The basis for representing these transformations is artificial neural network architectures. The CNN, which is used in this work, is a very successful deep learning architecture that has achieved state-of-the-art performance in image recognition tasks^51^ and more recently, has been used for the classification of time series data^55^.

Convolutional neural networks (CNN) have been inspired by the way the visual cortex in the human brain works, where local receptive fields react to visual stimuli located in a limited region of the visual field^56^. These receptive fields may overlap, tiling the whole visual field. CNNs implement this concept through two main building blocks: the convolutional layer and the pooling layer. The convolutional layer is formed by a series of neurons that are connected to neurons of a previous layer based on their receptive field. A first convolutional layer would be connected to a set of pixels of an input image within a receptive field (see convolution 1 in Fig. 3f), detecting features at a low level. Further convolutions would assemble these features into higher-level ones (see convolution 2 in Fig. 3f). The set of weights (i.e., filter) of a neuron in each convolution layer will depend on the type of feature it is “looking” for. As there will be multiple features at each level, each convolution layer is typically composed of more than one filter. Applying or convoluting these filters on the input or previous layers produces what is called feature maps which indicate the regions on the original input where the neurons were activated. These feature maps become the input to the following layer.

The pooling layer is another important building block which allows downscaling the output of the convolution, reducing dimensionality, sensitivity, and computational complexity. In general, a CNN architecture will consist of a set of convolutional and pooling layers that are stacked one after the other having the last layer connected to either a fully connected neural network or to any other machine learning structure to perform the final classification or prediction. In this work, a neural network with two fully connected layers and three output nodes has been implemented and connected to a two-convolution + pooling layer stack.

To train this architecture, the signals have been encoded through Gramian Angular Summation Fields (GASF) as images to take advantage of conventional, broadly used CNN’s. The encoding is performed in two steps. First, the time series is represented in a polar coordinate system. Given a time series X = x_1_, x_2_, …, x_n_ of n real-valued observations, X is normalized to the interval (1, − 1) and then each value is encoded as the angular cosine and the time stamp as the radius applying the following equations:

1 $$\varphi =arcos\left({x}_{i}\right),-1<{x}_{i}<1,{x}_{i}\in X$$

2 $$r={t}_{i}/N,{t}_{i}\in N$$

where t_i_ is the time stamp and N is a constant factor to regularize the span of the polar coordinate system. Figure 3d,e show an example of the polar coordinate representation of a raw signal and how is then encoded with the angular cosine.

The architecture used is shown in Fig. 3f, where the specific parameters of each layer are described. The total accuracy obtained from the test set was calculated simply by considering how many of the total tested observations were correct.

### Signal fitting

To produce the scatter plots in Fig. 2, the units of the raw time-resolved signal were converted from temporal to spatial using the sound velocity *ν*, extracted from the average Brillouin frequency (obtained through a fast Fourier transform) through the relationship:

3 $${f}_{B}=2n\nu /\lambda ,$$

where n is the refractive index, and $\lambda$ the probe wavelength (at normal incidence). The signal was then fitted to a decaying sinusoidal with the form:

4 $$TRBS=Asin\left(2\pi {f}_{B}t+\varphi \right){e}^{-{\alpha }_{0}t}$$

where *A* is the signal amplitude, φ is the phase, and α_0_ the sound attenuation coefficient.

### Cell culture

Coverslips were sterilized in 70% ethanol for 10 min and then washed 3 times on sterile PBS. The coverslips were treated with poly-l-lysine and left to dry in a cell culture incubator for 10 min. All cells came from ATCC: MDA-MB-231(HTB-26), MCF10a(CRL-10317), HeLa (CCL-2) and NIH/3T3(CRL-1658) were harvested, counted, and resuspended in DMEM + 10% FBS at a concentration of 20,000 cells/ml. The cells were then seeded directly onto the coverslips using 500 μl of the solution (or 10,000 cells) per coverslip and incubated in normal cell culture conditions (5% CO_2_, 32 °C) for 72 h. The cells were then fixed on coverslips by incubating in 2 × formalin (in PBS) for 30 min at room temperature. The cells were washed 3 times in sterile PBS before storing at 4° until required. For imaging, the coverslips were placed in a Life Cell Instruments Chamlide CF-T two-coverslip imaging chamber filled with water.

## Data Availability

All data, used in this paper will be available on request for purposes of reproducing or extending the analysis. Please send all enquiries to fernando.perez-cota@nottingham.ac.uk.
